# 3D Shapeable, Superior Electrically Conductive Cellulose Nanofibers/Ti_3_C_2_T_x_ MXene Aerogels/Epoxy Nanocomposites for Promising EMI Shielding

**DOI:** 10.34133/2020/4093732

**Published:** 2020-06-17

**Authors:** Lei Wang, Ping Song, Cheng-Te Lin, Jie Kong, Junwei Gu

**Affiliations:** ^1^Shaanxi Key Laboratory of Macromolecular Science and Technology, School of Chemistry and Chemical Engineering, Northwestern Polytechnical University, Xi'an, Shaanxi 710072, China; ^2^Key Laboratory of Marine Materials and Related Technologies, Zhejiang Key Laboratory of Marine Materials and Protective Technologies, Ningbo Institute of Materials Technology and Engineering (NIMTE), Chinese Academy of Sciences, Ningbo 315201, China

## Abstract

In this work, 3D highly electrically conductive cellulose nanofibers (CNF)/Ti_3_C_2_T_x_ MXene aerogels (CTA) with aligned porous structures are fabricated by directional freezing followed by freeze-drying technique, and the thermally annealed CTA (TCTA)/epoxy nanocomposites are then fabricated by thermal annealing of CTA, subsequent vacuum-assisted impregnation and curing method. Results show that TCTA/epoxy nanocomposites possess 3D highly conductive networks with ultralow percolation threshold of 0.20 vol% Ti_3_C_2_T_x_. When the volume fraction of Ti_3_C_2_T_x_ is 1.38 vol%, the electrical conductivity (*σ*), electromagnetic interference shielding effectiveness (EMI SE), and SE divided by thickness (SE/d) values of the TCTA/epoxy nanocomposites reach 1672 S m^−1^, 74 dB, and 37 dB mm^−1^, respectively, which are almost the highest values compared to those of polymer nanocomposites reported previously at the same filler content. In addition, compared to those of the samples without Ti_3_C_2_T_x_, the storage modulus and heat-resistance index of TCTA/epoxy nanocomposites are enhanced to 9792.5 MPa and 310.7°C, increased by 62% and 6.9°C, respectively, presenting outstanding mechanical properties and thermal stabilities. The fabricated lightweight, easy-to-process, and shapeable TCTA/epoxy nanocomposites with superior EMI SE values, excellent mechanical properties, and thermal stabilities greatly broaden the applications of MXene-based polymer composites in the field of EMI shielding.

## 1. Introduction

With the rapid development of modern electronic information technology, especially the aerospace weapons and equipment technology, electromagnetic interference (EMI) pollution caused by high-frequency and high-power electronic equipment is becoming more and more severe, and the request for EMI shielding performances of existing materials in service becomes increasingly demanding [1–4]. Investigations on the EMI shielding materials with low density, excellent mechanical properties, and remarkable shielding effectiveness (SE), especially, ease of large-scale and complex structure production, are of great significance for the development and upgrading of aerospace weapons and equipment.

Nowadays, conductive polymer nanocomposites are increasingly favored in the field of EMI shielding due to their high specific strength, adjustable properties, and excellent chemical stability [5–8]. Generally, it is necessary to add a large amount of conductive fillers to achieve the required EMI SE value (20 dB for commercial application), which usually causes processing difficulties and poor mechanical properties [9, 10]. In order to overcome this issue, researchers have attempted to further improve EMI SE values of the polymer composites by functionalized conductive fillers or microstructural regulation of the fillers. Eswaraiah et al. improved the dispersion of graphene in polyvinylidene fluoride (PVDF) by surface prefunctionalization of graphene sheets, and the EMI SE value of 7 wt% functionalized graphene/PVDF was up to 26 dB [11]. Yousefi et al. prepared graphene/epoxy nanocomposites by solution casting method, and the graphene/epoxy nanocomposites exhibited EMI SE value of 38 dB with a loading of 3 wt% graphene [12]. In our previous work, the EMI SE value of 35 wt% 3D graphene nanoplatelets/polystyrene (GNPs/PS) prepared by electrospinning method reached 33 dB, which was improved by 106% compared to that (16 dB) of randomly dispersed GNPs/PS at the same loading of GNPs [13]. The above methods can improve the possibility for the formation of conductive networks or decrease percolation threshold of conductive fillers. However, the improvement effect is limited based on the abovementioned methods, and it still demands a large amount of conductive fillers to achieve ideal EMI SE.

Construction of 3D conductive networks has proven to be an effective way to remarkably improve EMI SE values at low filler loading [14–17]. Chen et al. prepared carbon nanotube foam/epoxy nanocomposites by chemical vapor deposition (CVD) method. When the carbon nanotube foam was only 0.66 wt%, the EMI SE value of the nanocomposites was 33 dB, which was 10 times higher than that of the counterpart (0.66 wt%) prepared by blend-casting method, due to the formation of 3D highly conductive networks of carbon nanotube foam [18]. Sun et al. reported that PS@Ti_3_C_2_T_x_ nanocomposites fabricated by electrostatic self-assembly and molding method also demonstrated excellent EMI SE values, because of the contribution of 3D highly conductive networks embedded with the matrix [19]. With only 4.0 wt% Ti_3_C_2_T_x_, the electrical conductivity (*σ*) and EMI SE value of PS@Ti_3_C_2_T_x_ nanocomposites reached 1081 S m^−1^ and 54 dB, respectively. Although the EMI SE values based on 3D conductive networks are greatly increased compared to those of the composites prepared by blend-casting method with randomly oriented filler microstructure, they have the similar problems including energy-consuming, limited sample sizes, and uncontrollable shape. Recently, the porous foams made from the assembly of conductive fillers with the aid of polymers, such as cellulose nanofibers (CNF), polyvinyl alcohol (PVA), and polyetherimide (PEI), have been reported [20–25]. Ling et al. [26] prepared graphene/PEI hybrid foams with a pore size of 9-16 *μ*m by solvent evaporation, showing an adjustable EMI SE value between 3 and 13 dB by controlling the loading of graphene. Xu et al. prepared porous PVA/Ti_3_C_2_T_x_ hybrid foams with different thicknesses by freeze-drying and molding method after self-assembly of a mixture of PVA and Ti_3_C_2_T_x_ and realized the variable EMI SE values of 26-33 dB [27].

Although the 3D conductive networks with randomly distributed microstructure can significantly reduce the amount of conductive fillers and improve *σ* values, it still fails to make full use of conductive fillers to block electromagnetic waves. Compared to 3D conductive networks with isotropic orientation, aligned conductive networks can not only further reduce the percolation threshold of conductive fillers but also promote efficient utilization of filler/polymer interface, thus enhancing the multiple reflection and reabsorption of electromagnetic waves between aligned fillers and finally improving the attenuation of electromagnetic waves. Some techniques have been developed for the creation of aligned conductive networks, including template-derived method, directional freezing, and 3D printing process [28–30]. Compared to other methods, directional freezing is a simple, practical, and widely used method, which can be carried out in aqueous environment and easy to design the resulting structure. Li et al. prepared anisotropic graphene aerogel/epoxy nanocomposites with EMI SE value of 32 dB along axial direction by directional freezing and freeze-drying. Compared to conventional graphene aerogels (27 dB), EMI SE value of the nanocomposites was increased by 18.5% with 0.8 wt% graphene loading [31]. Zeng et al. reported 7.2 wt% anisotropic multiwalled carbon nanotubes (MWCNT)/waterborne polyurethane (WPU) foams with EMI SE value up to 50 dB along axial direction by directional freezing [32]. However, except graphene, so far the polymer nanocomposites with aligned conductive networks using other 2D materials, such as MXene (Ti_3_C_2_T_x_), have seldom been reported yet.

As a degradable nanomaterial with abundant source, CNF is a promising green material owing to its good biocompatibility, excellent mechanical properties, and low density and has broad application prospects in EMI shielding, energy storage, battery applications, etc. [33–36]. In this work, CNF is assembled with Ti_3_C_2_T_x_ nanosheets by hydrogen bonding, followed by directional freezing and freeze-drying technique, in order to obtain the CNF/Ti_3_C_2_T_x_ aerogel (CTA). The thermally annealed CTA (TCTA)/epoxy nanocomposites are then fabricated by thermal annealing of CTA, subsequent vacuum-assisted impregnation and curing method. The morphology and chemical compositions of Ti_3_C_2_T_x_ nanosheets and TCTA are characterized by scanning electron microscopy (SEM), X-ray diffraction (XRD), Raman spectroscopy, and X-ray photoelectron spectroscopy (XPS). Furthermore, the effect of the volume fraction of Ti_3_C_2_T_x_ nanosheets on *σ*, EMI SE values, mechanical properties, and thermal stabilities of TCTA/epoxy nanocomposites is investigated, and the mechanism of EMI SE improvement of the nanocomposites is also proposed.

## 2. Results

**Figure 1:**
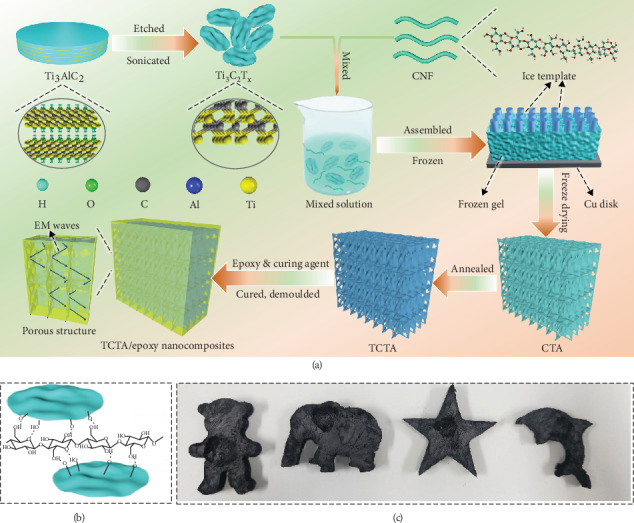
(a) Schematic illustration of fabrication for TCTA/epoxy nanocomposites. (b) Schematic illustration of the interaction between CNF and Ti_3_C_2_T_x_. (c) Digital images for various shapes of TCTA showing plasticity and machinability.

Figure 1(a) illustrates the schematic diagram of the fabrication of TCTA/epoxy nanocomposites. The Ti_3_C_2_T_x_ nanosheets are exfoliated by ion intercalation method and assembled with CNF by directional freezing and freeze-drying technique, followed by annealing to decrease the density and improve *σ*. Finally, the TCTA/epoxy nanocomposites are prepared by impregnation with epoxy resins. As indicated in Figure 1(b), CNF and Ti_3_C_2_T_x_ nanosheets overlap with each other by hydrogen bonding, which effectively prevents the agglomeration of Ti_3_C_2_T_x_ nanosheets. In Figure 1(c), TCTA with various complex shapes can be prepared *via* different molds, indicating that our proposed method can satisfy the fabrication of various complex structures with large-scale production.

**Figure 2:**
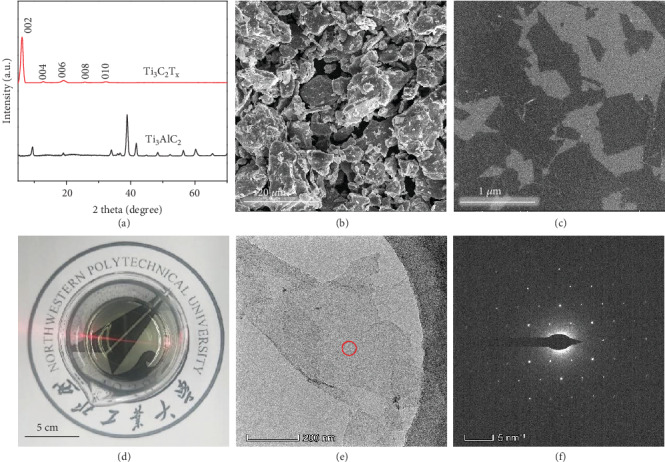
(a) XRD pattern for Ti_3_AlC_2_ and Ti_3_C_2_T_x_. SEM images of (b) Ti_3_AlC_2_ and (c) Ti_3_C_2_T_x_ nanosheets. (d) Tyndall effect of diluted Ti_3_C_2_T_x_ suspension using a laser pointer. (e) TEM image and (e′) SEAD pattern of Ti_3_C_2_T_x_ nanosheets.

Figure 2 shows the structural and morphological characterizations of Ti_3_C_2_T_x_ nanosheets. In Figure 2(a), the (002) diffraction peak shifts from 9.7° for Ti_3_AlC_2_ to 6.2° for Ti_3_C_2_T_x_, indicating that the corresponding interlayer spacing is enlarged from 1.10 to 1.42 nm, due to the removal of Al atomic layers, proving the successful synthesis of Ti_3_C_2_T_x_ nanosheets [37, 38]. In the wide-scan XPS spectra (Figure S1(b)), the Al 2s and Al 2p peaks of Ti_3_AlC_2_ disappear after etching, while the new peaks of O 1s and F 1s are observed in Ti_3_C_2_T_x_ [39], confirming the complete removal of Al atomic layer and the formation of -OH and -F groups on the surface of Ti_3_C_2_T_x_ nanosheets. In Figure S1(d), the absorption peaks at 3460, 1630, and 600 cm^−1^ of Ti_3_C_2_T_x_ nanosheets are ascribed to the stretching vibration from -OH, C=O, and Ti-O groups, respectively [40, 41]. In contrast to the dense-layered structures of Ti_3_AlC_2_ raw material in Figure 2(b) and Figure S1(a), after etching, Ti_3_C_2_T_x_ nanosheets exhibit typical 2D structure with uniform thickness and a lateral size of about 800 nm, as shown in Figure 2(c). Typical Tyndall effect appears in diluted Ti_3_C_2_T_x_ suspension in Figure 2(d), proving that the hydrophilic functional groups render the surface of Ti_3_C_2_T_x_ nanosheets to form a stable suspension. In Figure 2(e) and Figure S1(c), TEM and AFM images of Ti_3_C_2_T_x_ nanosheets indicate that the Ti_3_C_2_T_x_ nanosheets are ultrathin with an average thickness of 2.04 nm and highly transparent under electron irradiation, in which the surface of Ti_3_C_2_T_x_ nanosheets is clear without any impurity and byproduct. In addition, the selected-area electron diffraction (SEAD) pattern image (Figure 2(e′)) reveals the typical hexagonal structure of Ti_3_C_2_T_x_ nanosheets [42]. All the results demonstrate the successful synthesis of Ti_3_C_2_T_x_ nanosheets with good crystallinity and clean surface.

**Figure 3:**
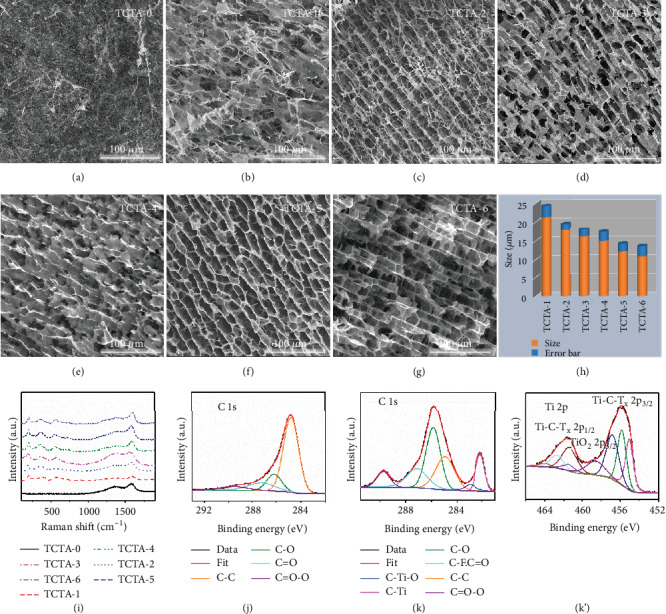
(a–g) SEM images of TCTA. (h) Comparison of average cell sizes for TCTA. (i) Raman spectra of TCTA. (j) C 1s spectra of TCTA-0. (k) C 1s and (k′) Ti 2p spectra of TCTA-6.

Figures 3(b)–3(g) present the SEM images of TCTA-(1-6) prepared by directional freeze-drying process using Ti_3_C_2_T_x_ nanosheets and CNF, in which Ti_3_C_2_T_x_ and CNF support each other, thus preventing agglomeration of each component and promoting the formation of aligned porous structures which would not be altered after annealing. In contrast, TCTA-0 exhibits a dense porous structure with random orientation based on the intertwining of CNF (Figures 3(a) and S2). As shown in Figures 3(h) and S3, with increasing volume fraction of Ti_3_C_2_T_x_ nanosheets, the cell density of TCTA monotonically increases and the cell size correspondingly decreases from 21.3 *μ*m (TCTA-1) to 10.2 *μ*m (TCTA-6). The aligned porous structures can be attributed to the fact that ice crystals grow from the bottom to the top of the samples during directional freezing process, by which Ti_3_C_2_T_x_ and CNF are forced to align along the growth direction of ice crystals, resulting in the formation of highly aligned microstructure after freeze-drying. And the cell size decreases with an increase of volume fraction of Ti_3_C_2_T_x_ which is attributed to the increased nucleation density of active seeds that limits the further growth of ice crystals. In Figure 3(i), the characteristic peaks in the Raman spectra of TCTA for D- and G-bands at 1352 and 1584 cm^−1^, respectively, are attributed to the residual carbon from decomposed CNF after annealing [43–45]. After incorporation of Ti_3_C_2_T_x_, the newly appeared peak at 200 cm^−1^ can be assigned to A1g symmetry out-of-plane vibration of Ti atoms, and the peaks at 374 and 583 cm^−1^ are ascribed to the Eg group vibration and in-plane (shear) modes of Ti, C, and surface functional groups [46]. In Figure S4, there are two peaks ascribed to Ti and F elements in the XPS survey spectrum of TCTA-6 [47] but cannot be seen in the case of TCTA-0. Moreover, compared to the C 1s spectrum of TCTA-0 (Figures 3(j) and 3(k)) (TCTA-6) presents Ti-C (282.3 eV), Ti-C-O (283.2 eV), and C-F (287 eV) bonds due to the incorporation of Ti_3_C_2_T_x_ nanosheets. In addition, the ratio of characteristic peaks of C-O and C=O-O still maintains a high level after annealing. It indicates that there are still a large number of polar functional groups attributed to Ti_3_C_2_T_x_ and CNF in TCTA-6, such as -OH and –COOH. The characteristic peak of TiO_2_ can be observed in the Ti 2p XPS spectrum of TCTA-6 in Figure 3(k′) owing to the weak oxidation of Ti_3_C_2_T_x_ (Figure S5) during etching process.

**Figure 4:**
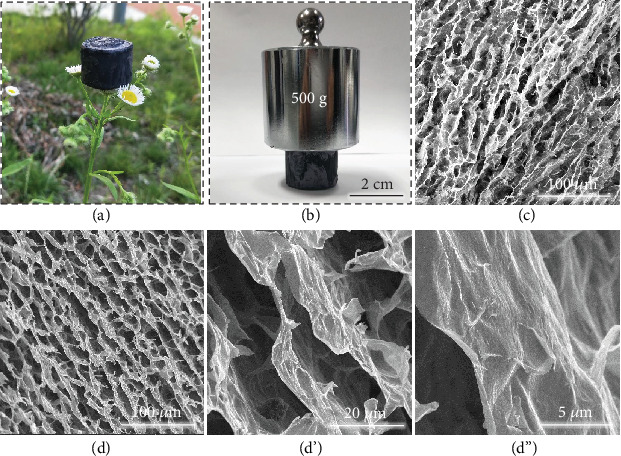
(a) Digital images of TCTA-6 standing on a flower and (b) supporting a load of 500 g. (c) Top view and (d-d^″^) side view SEM images of TCTA-6.

The aerogel sample (TCTA-6) has a lightweight architecture (Figure 4(a)) and excellent mechanical properties to support a 500 g load (1300 times larger than the weight itself (390 mg), see Figure 4(b)), which is based on the well-aligned porous structure and reinforcement of CNF. The sample density ranges from 2.4 mg cm^−3^ (TCTA-0) to 45.0 mg cm^−3^ (TCTA-6), as listed in Table S1. In Figures 4(c)–4(d^″^), both top and cross-sectional views of TCTA-6 exhibit similar aligned porous structure. In these SEM images, Ti_3_C_2_T_x_ nanosheets overlap with each other to form rigid and long-range aligned cell walls, giving TCTA-6 an outstanding mechanical property to resist compression, and the cells composed of Ti_3_C_2_T_x_ nanosheets can serve as a buffer against stress.

**Figure 5:**
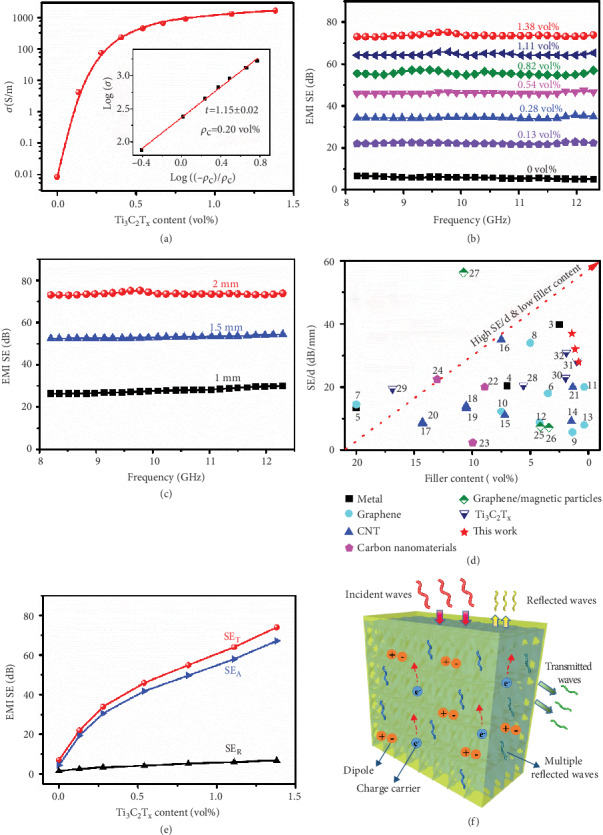
(a) *σ* values of the TCTA/epoxy nanocomposites. (b) EMI SE values of the TCTA/epoxy nanocomposites at X-band. (c) Thickness *vs.* EMI SE values of the TCTA-6/epoxy nanocomposites. (d) Comparison of EMI SE values for TCTA-6/epoxy nanocomposites (marked as red stars) with those of other reported works. SE/d is depicted as a function of conductive filler volume fraction. Numbers inside the plot correspond to the reference information listed in Table S3. (e) SE_T_, SE_A_, and SE_R_ values of the TCTA/epoxy nanocomposites. (f) Schematic diagram of the shielding mechanism for TCTA/epoxy nanocomposites.

The electrical conductivities (*σ*) and EMI SE values of TCTA and corresponding nanocomposites were investigated as the results shown in Figure 5. In Figures 5(a) and S6(a) and Tables S1 and S2, the *σ* values of TCTA increase rapidly from 0.0186 S m^−1^ to 1992 S m^−1^ for TCTA-0 and TCTA-6, respectively, and the corresponding *σ* values of the nanocomposites increase from 8.6 × 10^−3^ S m^−1^ to 1672 S m^−1^ for TCTA-0/ and TCTA-6/epoxy nanocomposites with 1.38 vol% Ti_3_C_2_T_x_ addition, respectively. As the volume fraction of Ti_3_C_2_T_x_ increases, the contact possibility between Ti_3_C_2_T_x_ nanosheets with excellent electrical conductivity increases within the matrix, resulting in the formation of 3D conductive networks in TCTA/epoxy nanocomposites with excellent *σ* values [48]. The TCTA-6/epoxy nanocomposites present anisotropic electrical conductivity, which is 1672 S m^−1^ along the axial direction and 1283 S m^−1^ along the radial direction (see Figure S6(b)). Our results exhibit that the *σ* values of TCTA/epoxy nanocomposites are in accordance with the power law equation:

(1) $$\begin{array}{c} \sigma =\sigma_{0}{\left[\left(\rho -\rho_{\text{c}}\right)\left(1-\rho_{\text{c}}\right)\right]}^{t}, \end{array}$$

where *σ*_0_ is a constant dependent on the intrinsic conductivity of Ti_3_C_2_T_x_ nanosheets, *ρ*_c_ is the volume fraction of Ti_3_C_2_T_x_ at percolation threshold, and *t* is the critical exponent reflecting the dimensionality of the nanocomposites. The fitting results show the percolation threshold is 0.20 vol% of Ti_3_C_2_T_x_ (Figure 5(a)), which is much lower than 1.70 vol% in Ti_3_C_2_T_x_/PAM [49], 0.60 vol% in rGO/PVA [50], and also slightly lower than 0.26 vol% in PS@Ti_3_C_2_T_x_ [19]. Moreover, the *σ* value (1672 S m^−1^) of TCTA/epoxy nanocomposites is almost the highest value in comparison with previously reported polymer composites with conductive fillers such as Ti_3_C_2_T_x_, graphene, and carbon nanotubes, at the same filler content [51, 52]. The obtained *σ* value is also obviously higher than that of recently reported 3D graphene/Ti_3_C_2_T_x_ aerogel/epoxy nanocomposite (695.9 S m^−1^) at 0.99 vol% graphene/Ti_3_C_2_T_x_ content [52] and that of PS@rGO (1083 S m^−1^) at 4.80 vol% rGO content [53]. The above results indicate that highly conductive Ti_3_C_2_T_x_ and developed conductive networks endow TCTA/epoxy nanocomposites with efficient electron transportation capability, thus presenting remarkable *σ* values.

Figure 5(b) illustrates EMI SE values of TCTA/epoxy nanocomposites at X-band. The EMI SE value of the nanocomposites without Ti_3_C_2_T_x_ addition (TCTA-0) is only 7 dB. After incorporation of Ti_3_C_2_T_x_, the EMI SE value of TCTA-1/epoxy nanocomposites at 0.13 vol% Ti_3_C_2_T_x_ content rises rapidly to 22 dB (commercial requirement: 20 dB). When the volume fraction of Ti_3_C_2_T_x_ is 1.38 vol%, the EMI SE value of TCTA-6/epoxy nanocomposites approaches 74 dB, which is slightly lower than that of TCTA-6 without epoxy matrix (Figure S8(a)), but significantly higher than that of the nanocomposites prepared by blend-casting method with the same volume fraction of Ti_3_C_2_T_x_ (19 dB), as displayed in Figure S8(b). In addition, the EMI SE value of TCTA-6/epoxy nanocomposites along the radial direction (74 dB) is much higher than that along the axial direction (57 dB, Figure S8(c)), presenting anisotropic EMI shielding behavior. The *σ* values of TCTA/epoxy nanocomposites increase gradually with increasing volume fraction of Ti_3_C_2_T_x_, causing stronger electric loss (eddy current loss) and enhanced EMI shielding performance.

The specimen thickness is another key factor influencing the EMI SE values of TCTA/epoxy nanocomposites. As an example, TCTA-6/epoxy nanocomposites with 1 mm in thickness display EMI SE value of 30 dB (Figure 5(c)), which is rapidly increased to 74 dB for 2 mm thick sample. In order to further highlight the advantages of highly efficient 3D conductive networks of TCTA/epoxy nanocomposites, the EMI SE divided by thickness (SE/d) reported in the literatures are summarized in Figure 5(d) and Table S3 to compare with our results. Some recent works can be seen in Figure 5(d), such as 10 dB mm^−1^ for 1.34 vol% CNT sponge/epoxy nanocomposites [18], 20 dB mm^−1^ for 0.36 vol% graphene aerogel/polydimethylsiloxane (PDMS) [54], and 31 dB mm^−1^ for 1.9 vol% PS@Ti_3_C_2_T_x_ [19]. In contrast, our TCTA/epoxy nanocomposites achieve higher SE/d of 28, 32, and 37 dB mm^−1^ at lower loading of Ti_3_C_2_T_x_ (0.82, 1.11, and 1.38 vol%), respectively, demonstrating superior EMI shielding performances.

**Figure 6:**
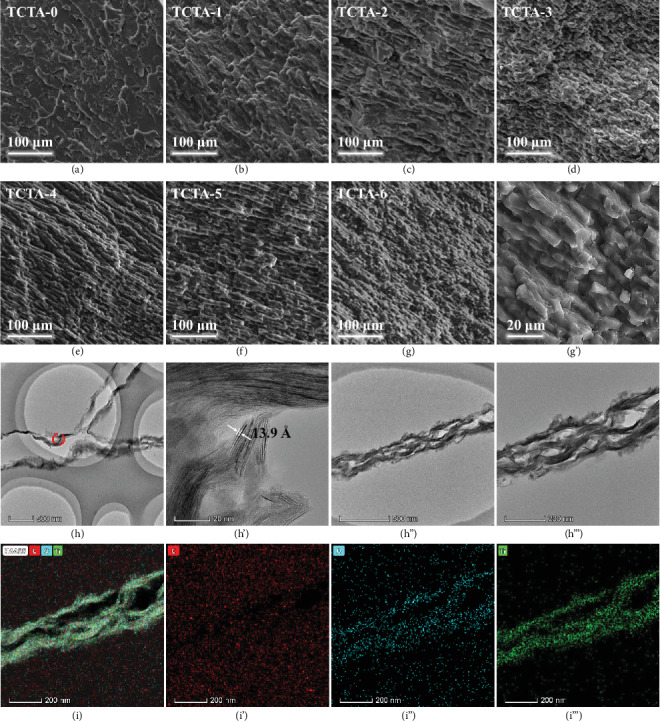
Side view SEM images of the epoxy nanocomposites of (a) TCTA-0, (b) TCTA-1, (c) TCTA-2, (d) TCTA-3, (e) TCTA-4, (f) TCTA-5, (g, g′) TCTA-6, and (h′) HRTEM and (h–h^‴^) STEM images of the TCTA-6/epoxy nanocomposites and the corresponding (i) HAADF image, elemental mapping images of (i′) C, (i^″^) O, and (i^‴^) Ti at the same area.

To explore the shielding mechanism of TCTA/epoxy nanocomposites, Figure 5(e) compares the contribution of absorption shielding effectiveness (SE_A_) and reflection shielding effectiveness (SE_R_) to total shielding effectiveness (SE_T_). Consistent with SE_T_, SE_A_ increases dramatically and is much higher than SE_R_. This is because the integrity of aligned porous structures of TCTA is well-maintained inner TCTA/epoxy nanocomposites (Figures 6(a)–6(g′)). In the scanning transmission electron microscope (STEM) images of TCTA-6/epoxy nanocomposites (Figures 6(h)–6(h^‴^)), a large number of Ti_3_C_2_T_x_ nanosheets overlap with each other to form hollow-like skeleton structures, which still remain in the TCTA-6/epoxy nanocomposites after impregnating with epoxy resins. Ti_3_C_2_T_x_ nanosheets are assembled into the backbone of the porous structures without any individual nanosheets randomly dispersed inner epoxy matrix. It can be seen from the HRTEM image (Figure 6(h′)) that intrinsic structures of Ti_3_C_2_T_x_ are not destroyed after impregnating with epoxy resins, and the interlayer spacing maintains 13.9 Å, similar to that of previous XRD (Figure 2(a)) results, indicating that Ti_3_C_2_T_x_ is effectively protected by epoxy resin from oxidation. It is especially important to note that, according to the corresponding high-angle annular dark field image (HAADF, Figure 6(i)) and C elemental spectrum (Figure 6(i′)), the interior of the backbone of TCTA-6 is not hollow structures, but the carbon-rich area. Combined with the elemental distribution of O and Ti elements (Figures 6(i^″^) and 6(i^‴^)) by EDS mapping, it proves that the outer parts of the framework are mainly compactly attached by Ti_3_C_2_T_x_ nanosheets, and CNF is encapsulated. This is mainly due to the fact that a large number of Ti_3_C_2_T_x_ nanosheets interweave in cross-linking CNF networks and wrap on CNF and Ti_3_C_2_T_x_ nanosheets which overlap with each other, promoting the construction of highly conductive networks. The porous structures of TCTA improve the impedance matching characteristics and facilitate electromagnetic waves to enter the interior of TCTA/epoxy nanocomposites. When electromagnetic waves strike on TCTA/epoxy nanocomposites, a portion of the electromagnetic waves is reflected due to impedance mismatch. Transmitted electromagnetic waves will further be converted into thermal energy due to strong electric loss by interaction between electromagnetic waves and massive microcurrents in conductive networks [55]. Unique porous structures own large specific surface area and abundant interfaces. Due to interfacial polarization, local capacitive-like structures are induced by a large number of dipoles, enhancing the dielectric loss to electromagnetic waves. Moreover, the porous structures prolong the transmission paths of the electromagnetic waves by multiple reflection or scattering and further promote the reabsorption of the electromagnetic waves, which enhance the EMI SE values. In addition, the surface groups and defects of TCTA are beneficial to the attenuation of the electromagnetic waves owing to strengthened space charge polarization under the alternating electromagnetic field. The schematic diagram of the shielding mechanism for TCTA/epoxy nanocomposites is illustrated in Figures 5(f) and S9. Therefore, the absorption plays the key role in shielding mechanism for TCTA/epoxy nanocomposites.

**Figure 7:**
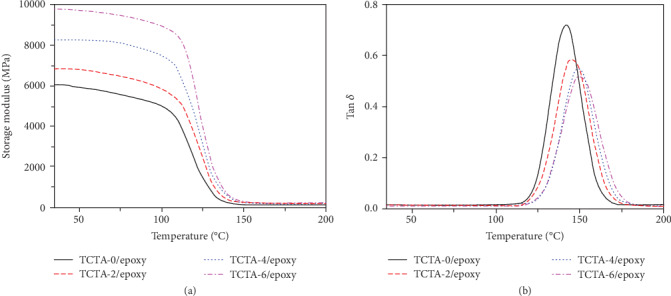
(a) Storage modulus *vs.* temperature and (b) Tan*δvs.* temperature of the TCTA/epoxy nanocomposites.

Figure 7 shows the DMA curves of TCTA/epoxy nanocomposites. In Figure 7(a), as the temperature increases, the nanocomposites gradually change from the glassy state to the elastic state with a decrease of the storage modulus. The storage modulus of TCTA-0/epoxy nanocomposites in the glassy state is only 6039.0 MPa, whereas the storage modulus of TCTA-6/epoxy nanocomposites (1.38 vol% Ti_3_C_2_T_x_) reaches 9792.5 MPa with an improvement of 62%. In addition, the glass transition temperature (*T*_g_) also increases from 141.9°C to 150.3°C for TCTA-0/ and TCTA-6/epoxy nanocomposites, respectively, due to the strong interaction between Ti_3_C_2_T_x_ and epoxy. TGA results in Figure S10 and Table S4 indicate that the residues of TCTA/epoxy nanocomposites gradually grow with an increase of the volume fraction of Ti_3_C_2_T_x_, and the corresponding *T*_Heat−resistance index_ (*T*_HRI_) increases from 303.8°C to 310.7°C for TCTA-0/ and TCTA-6/epoxy nanocomposites [56], respectively, demonstrating that the incorporation of Ti_3_C_2_T_x_ can enhance the thermal stabilities of the nanocomposites.

## 3. Discussion

3D highly conductive TCTA are assembled by directional freezing and freeze-drying method. After impregnating with epoxy resins, percolation threshold of Ti_3_C_2_T_x_ in TCTA/epoxy nanocomposites is only 0.20 vol%. And when the volume fraction of Ti_3_C_2_T_x_ is 1.38 vol%, the *σ*, EMI SE, and SE/d values of the TCTA-6/epoxy nanocomposites reach 1672 S/m, 74 dB, and 37 dB mm^−1^, respectively, almost the optimal results compared to those of previously reported polymer nanocomposites. Owing to highly conductive porous networks, absorption dominates the shielding mechanism. In addition, the storage modulus and *T*_HRI_ values of TCTA-6/epoxy nanocomposites are enhanced to 9792.5 MPa and 310.7°C, increased by 62% and 6.9°C, respectively, compared to those of TCTA-0/epoxy nanocomposites. Our fabricated lightweight, easy-to-process, and shapeable TCTA/epoxy nanocomposites with superior EMI shielding performance, mechanical properties, and thermal stabilities will greatly widen the applications of MXene nanomaterials and epoxy resins in the field of high-tech EMI shielding.

## 4. Materials and Methods

Experimental details including Ti_3_C_2_T_x_ synthesis, fabrication of TCTA and TCTA/epoxy nanocomposites, and characterizations can be found in Supplementary 1.

## Supplementary Materials

Supplementary Materials Supplementary 1. Figure S1: SEM image and wide-scan XPS spectrum of Ti_3_AlC_2_; wide-scan XPS spectrum, AFM image, and FTIR spectrum of Ti_3_C_2_T_x_. Figure S2: SEM image of TCTA-0. Figure S3: cell size and distribution of TCTA. Figure S4: wide-scan XPS spectra of TCTA-0 and TCTA-6. Figure S5: Ti 2p spectra of Ti_3_C_2_T_x_. Figure S6: *σ* values for TCTA and TCTA/epoxy nanocomposites. Figure S7: digital photographs showing LED lamp with TCTA-6/epoxy nanocomposites. Figure S8: EMI SE values of TCTA-6, TCTA-6/epoxy nanocomposites, and epoxy nanocomposites fabricated by blend-casting method. Figure S9: interaction between electromagnetic waves and TCTA/epoxy nanocomposites. Figure S10: TGA curves of the TCTA/epoxy nanocomposites. Table S1: comparison of electrical conductivities for TCTA with different densities. Table S2: comparison of EMI SE values for TCTA/epoxy nanocomposites. Table S3: comparison of EMI SE values of the TCTA/epoxy nanocomposites with other works. Table S4: thermal parameters of the TCTA/epoxy nanocomposites.
