# Supplementary material for: 3D Shapeable, Superior Electrically Conductive Cellulose Nanofibers/Ti3C2Tx MXene Aerogels/Epoxy Nanocomposites for Promising EMI Shielding
Source: Research (Wash D C). 2020 Jun 17;2020:4093732. doi: 10.34133/2020/4093732 (PMC7317662; doi:10.34133/2020/4093732)
Supplement: Supplementary Materials — Supplementary 1. Figure S1: SEM image and wide-scan XPS spectrum of Ti3AlC2; wide-scan XPS spectrum, AFM image, and FTIR spectrum of Ti3C2Tx. Figure S2: SEM image of TCTA-0. Figure S3: cell size and distribution of TCTA. Figure S4: wide-scan XPS spectra of TCTA-0 and TCTA-6. Figure S5: Ti 2p spectra of Ti3C2Tx. Figure S6: σ values for TCTA and TCTA/epoxy nanocomposites. Figure S7: digital photographs showing LED lamp with TCTA-6/epoxy nanocomposites. Figure S8: EMI SE values of TCTA-6, TCTA-6/epoxy nanocomposites, and epoxy nanocomposites fabricated by blend-casting method. Figure S9: interaction between electromagnetic waves and TCTA/epoxy nanocomposites. Figure S10: TGA curves of the TCTA/epoxy nanocomposites. Table S1: comparison of electrical conductivities for TCTA with different densities. Table S2: comparison of EMI SE values for TCTA/epoxy nanocomposites. Table S3: comparison of EMI SE values of the TCTA/epoxy nanocomposites with other works. Table S4: thermal parameters of the TCTA/epoxy nanocomposites. [file 4093732.f1.docx]

**3D Shapeable, Superior Electrically Conductive Cellulose Nanofibers/Ti_3_C_2_T_x_ MXene Aerogels/Epoxy Nanocomposites for Promising EMI Shielding**

Lei Wang^1^, Ping Song^1^, Cheng-Te Lin^2^, Jie Kong^1^ and Junwei Gu^1^*

^1^ Shaanxi Key Laboratory of Macromolecular Science and Technology, School of Chemistry and Chemical Engineering, Northwestern Polytechnical University, Xi’an, Shaanxi, 710072, P. R. China.

^2^ Key Laboratory of Marine Materials and Related Technologies, Zhejiang Key Laboratory of Marine Materials and Protective Technologies, Ningbo Institute of Materials Technology and Engineering (NIMTE), Chinese Academy of Sciences, Ningbo 315201, P.R. China

Corresponding author, E-mail: gjw@nwpu.edu.cn or nwpugjw@163.com (J. Gu)

**S1.1. Main Materials**

Ti_3_AlC_2_ powder (38 μm, 98% purity) was supplied by 11 technology Co., Ltd. (Jilin, China). Concentrated HCl and LiF were both bought from Macklin (Shanghai Co., China). Cellulose nanofibers (CNF, 4-10 nm in diameter and 1-3 μm in length) were received from Qihong technology Co., Ltd. (Guangxi Co., China). Bisphenol F epoxy (Epon 862) and diethyl methyl benzenediamine were provided by Hexion Inc (Columbus Co., USA) and Baiduchem Co., Ltd. (Hubei, China), respectively.

**S1.2. Fabrication of Ti_3_C_2_T_x_ nanosheets**

Ti_3_C_2_T_x_ nanosheets were firstly synthesized by modified minimally intensive layer delamination (MILD) method as reported^[1]^. Etching solution was prepared by dissolving 1.6 g LiF in 20 mL HCl (9M). Then 1.0 g Ti_3_AlC_2_ powder was gradually added into the above mixed solution in an ice bath within 5 min, which was then stirred at 500 rpm and kept reaction at 35^o^C for 24 hrs. Subsequently, the obtained products were centrifuged with deionized water at 3500 rpm for 5 min for each cycle until pH was close to neutral, finally to become the dark-green supernatant. Then the Ti_3_C_2_T_x_ sediment was dispersed in 100 mL of deionized water and centrifuged at 3500 rpm for 2 min, to separate the unreacted Ti_3_AlC_2_. The dark concentrated supernatant of Ti_3_C_2_T_x_ nanosheets was then sonicated by a probe sonicator (300 W) for 5 min. Finally, the few-layered Ti_3_C_2_T_x_ could be obtained by centrifugation at 3500 rpm for 1 hr, followed by freeze-drying of the supernatant solution.

**S1.3. Fabrication of thermally annealed CNF/Ti_3_C_2_T_x_ aerogels (TCTA)**

Different amounts of Ti_3_C_2_T_x_ nanosheets were dispersed in 10 mL cellulose nanofiber solution with a concentration of 2.5 mg mL^-1^ in a glass vessel by a probe sonication for 10 min in an ice bath, followed by vigorous stirring for 3 hrs. Then the cylindrical glass vessel was put on a pre-cooled copper plate placed on the surface of liquid nitrogen, to freeze the solution directionally. CNF/Ti_3_C_2_T_x_ aerogel (CTA) was obtained by freeze-drying at -60^o^C with a background pressure less than 5 Pa, followed by annealing at 400°C for 2 hrs at a heating rate of 5°C s^-1^ in an Ar (5% H_2_) atmosphere, to obtain thermally annealed CTA (TCTA). The content of Ti_3_C_2_T_x_ in CNF/Ti_3_C_2_T_x_ aerogels was 50, 100, 200, 300, 400, and 500 mg, respectively, and the corresponding aerogel was marked as TCTA-1, TCTA-2, TCTA-3, TCTA-4, TCTA-5, and TCTA-6. The CNF aerogel without the addition of Ti_3_C_2_T_x_ nanosheets was also prepared and named as TCTA-0 for comparison.

**S1.4. Fabrication of the TCTA/epoxy nanocomposites**

Epon 862 and diethyl methyl benzene diamine were stirred at 70 for 1 hr, and then filled into the above TCTA *via* vacuum-assisted impregnation technique. Finally, the TCTA/epoxy nanocomposites were prepared by curing at 120°C for 5 hrs. The average weight of TCTA-0 was measured as 7.3 mg, and the residual mass of CNF after annealing in different samples was regarded as the same as that of TCTA-0. The fraction of Ti_3_C_2_T_x_ and CNF was calculated on the basis of the density by following equations:

vol% (Ti_3_C_2_T_x_) = wt% (Ti_3_C_2_T_x_) × ρ_A_/ρ_M_ (Equation S1)

ρ_A_ = m_A_/V_A_ (Equation S2)

m_M_ = m_A_-m_c_ (Equation S3)

Where, m_A_, V_A_, and ρ_A_ were the mass, volume, and density of TCTA samples, respectively, m_M_, wt% (Ti_3_C_2_T_x_), and vol% (Ti_3_C_2_T_x_) were the mass, mass fraction, and volume fraction of Ti_3_C_2_T_x_ in TCTA, respectively, m_c_ was the mass of CNF in TCTA. The density of Ti_3_C_2_T_x_ was 3.2 g cm^-3^ as reported,^[2]^ and density of thermally annealed CNF was measured as 1.8 g cm^-3^.

**S1.5. Characterizations**

X-ray diffraction (XRD) of the samples was tested on a Shimadzu-7000 type X-ray diffraction (Shimadzu, Japan, *λ* = 0.154 nm). X-ray photoelectron spectroscopy (XPS) analyses of the samples were collected on a PHI5400 equipment (PE Corp., England). Raman spectra of the samples were measured on a WITec Alpha300R (PE Corp., England) with a He-Ne laser, tuned at 532 nm. Fourier transform infrared (FTIR) spectroscopy of the samples were carried out on a Bruker Tensor 27 device (Bruker Co., Germany) with thin films on KBr. Thermogravimetric analyses (TGA) of the samples were carried out using STA 449F3 (Netzsch C Corp., Germany) at 10^o^C min^-1^ at argon atmosphere over the temperature range of 40-800^o^C. Dynamic mechanical analyses (DMA) of the samples were performed by DMA/SDTA861e (METTLER TOLEDO Corp., Switzerland) with a frequency of 1 Hz and a heating rate of 5^o^C min^-1^ in the temperature range of 35-200^o^C, and the corresponding specimen dimension was of 50 × 10 × 4 mm. Scanning electron microscopy (SEM) images of the samples were captured on a VEGA3-LMH equipment (TESCAN Co., Czech Republic). Transmission electron microscopy (TEM) images of the samples were obtained on a Talos F200X/TEM microscope (FEI Co., USA) operated at 200 kV. Atomic force microscopy (AFM) images of the samples were collected by a Dimension Fast Scan AFM (Bruker Co., USA). Electrical conductivities (σ) values of the samples were analyzed using RTS-8 (Guangzhou Four Probes Technology Corp., China). EMI shielding performances of the samples were measured by an MS4644A Vector Network Analyzer instrument (Anritsu Corp., Japan), which used the wave-guide method at X-band frequency range according to ASTMD5568-08, and the specimen dimension was 22.86 × 10.16 × 2.00 mm.


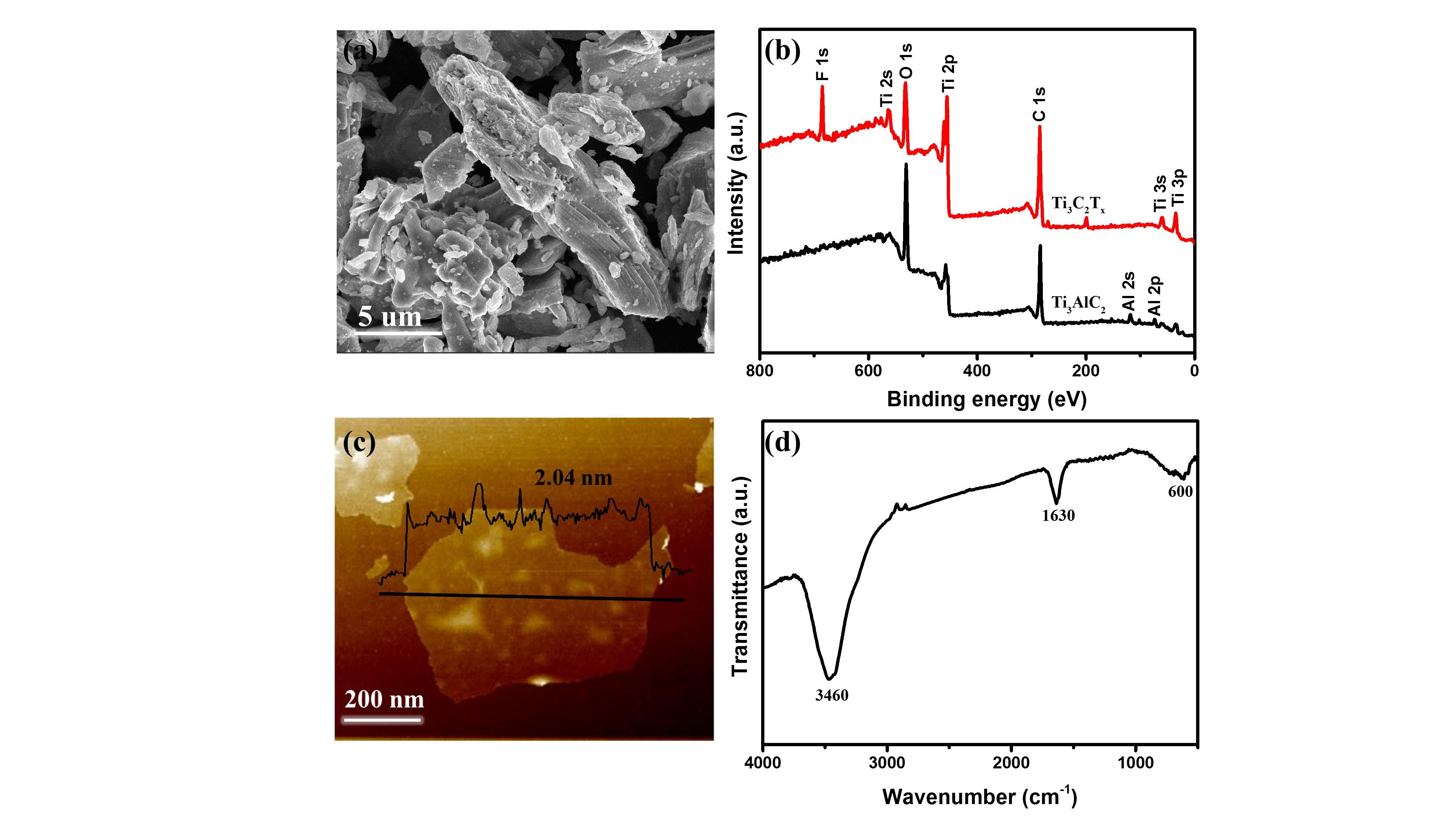


**Figure S1.** (a) SEM image of Ti_3_AlC_2_; (b) Wide-scan XPS spectra of Ti_3_AlC_2_ and Ti_3_C_2_T_x_; (c) AFM image of Ti_3_C_2_T_x_; (d) FTIR spectrum of Ti_3_C_2_T_x_.

**
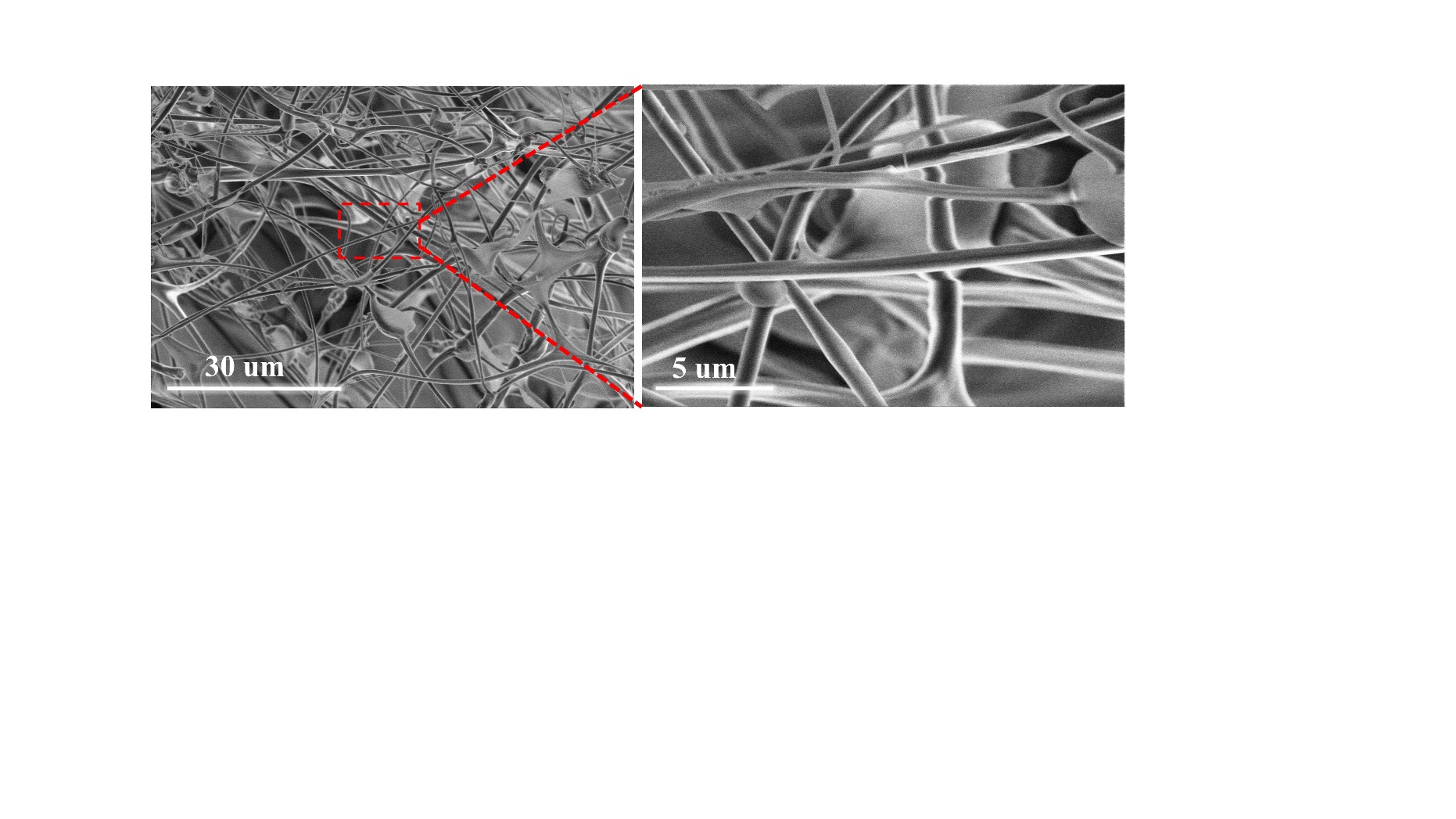
**

**Figure S2.** SEM images of TCTA-0.


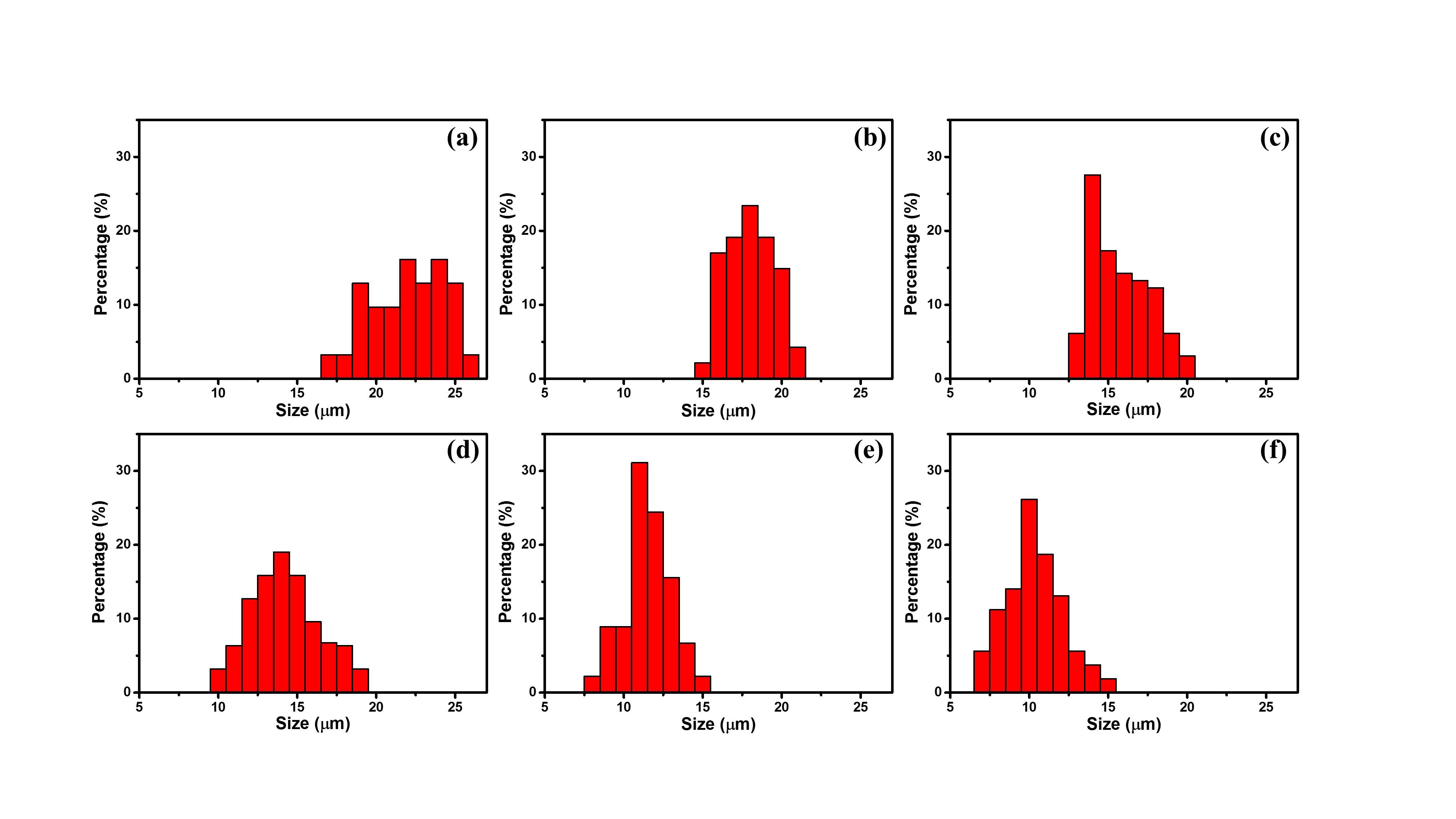


**Figure S3.** Cell size and distribution of (a) TCTA-1, (b) TCTA-2, (c) TCTA-3, (d) TCTA-4, (e) TCTA-5, and (f) TCTA-6.

**
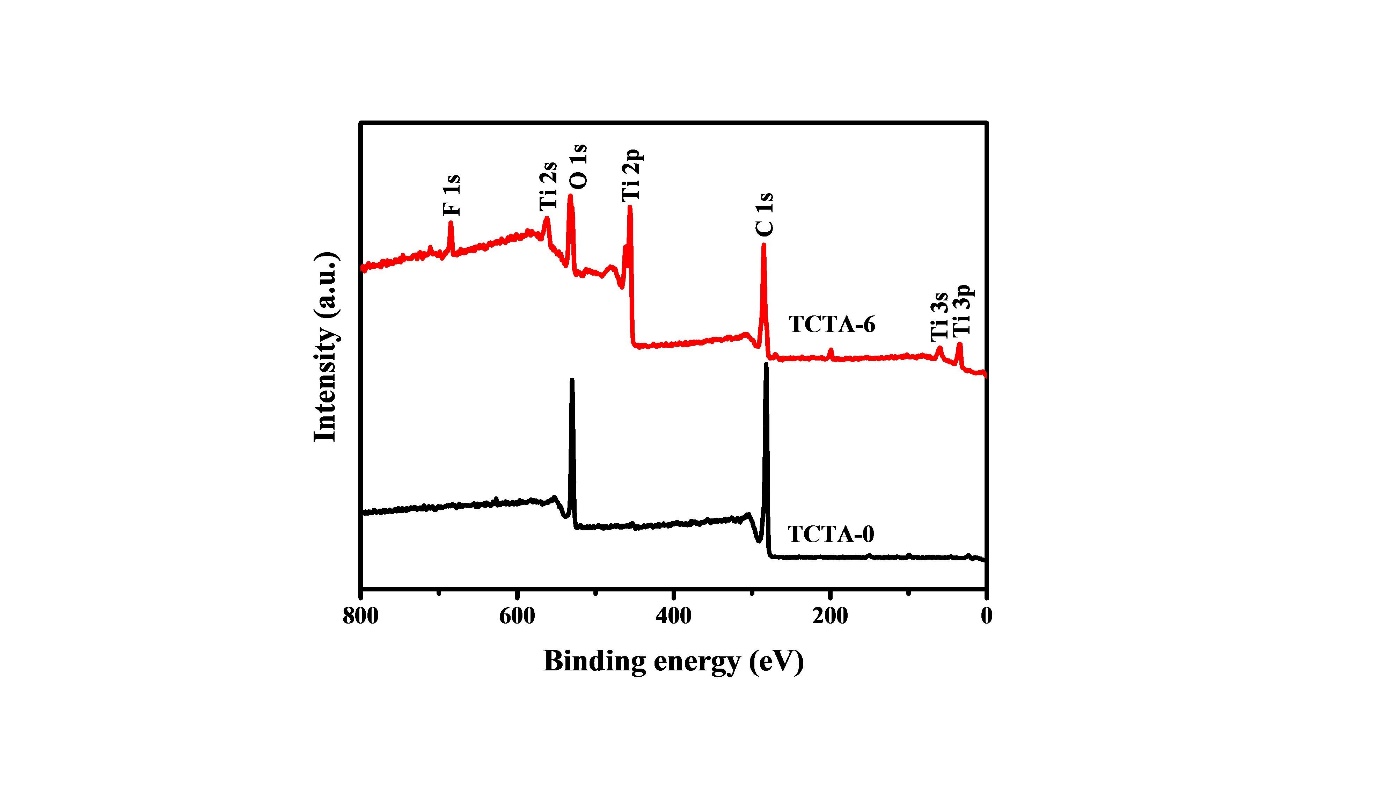
**

**Figure S4.** Wide-scan XPS spectra of TCTA-0 and TCTA-6.

**
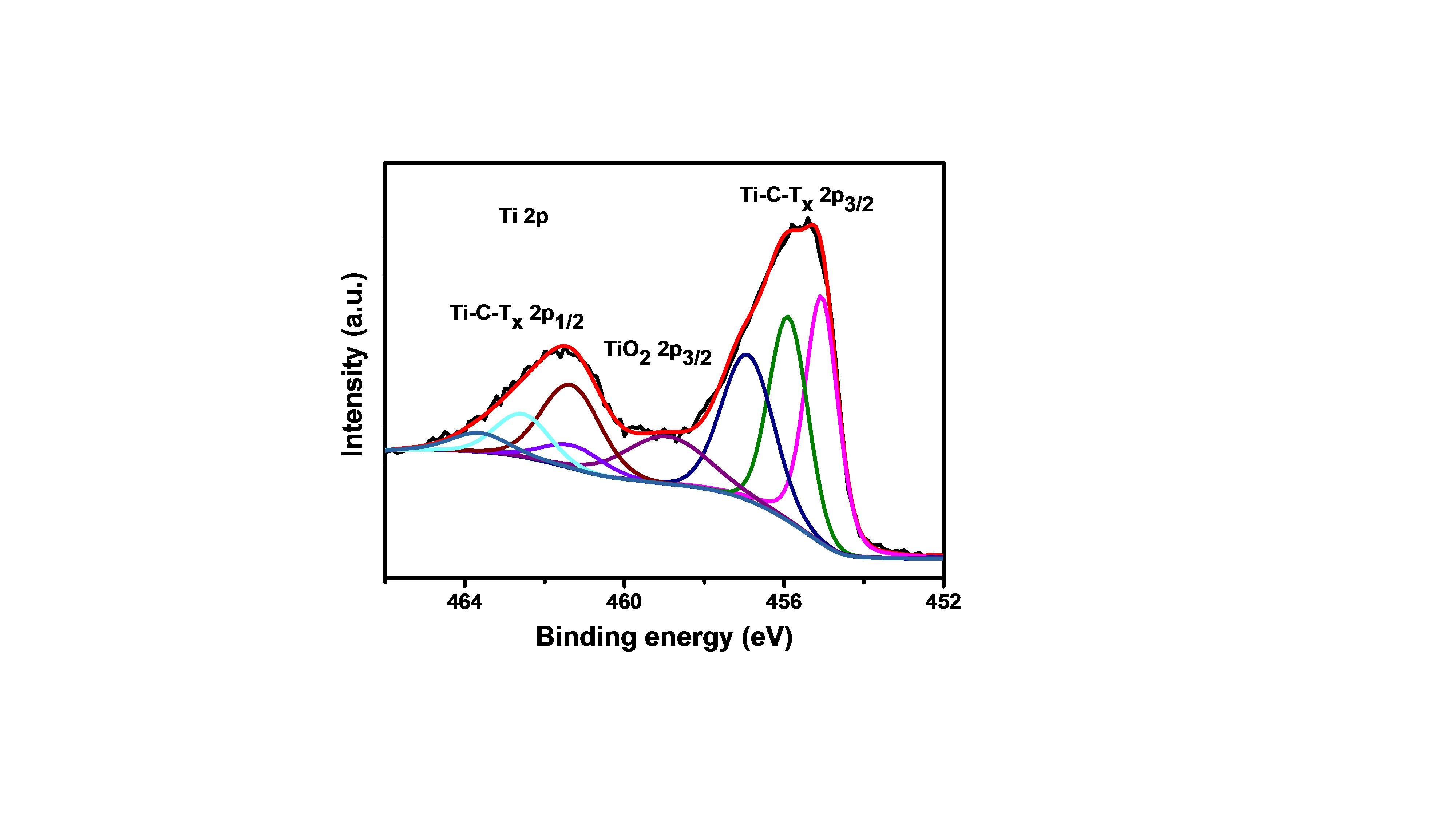
**

**Figure S5.** Ti 2p spectra of Ti_3_C_2_T_x_.


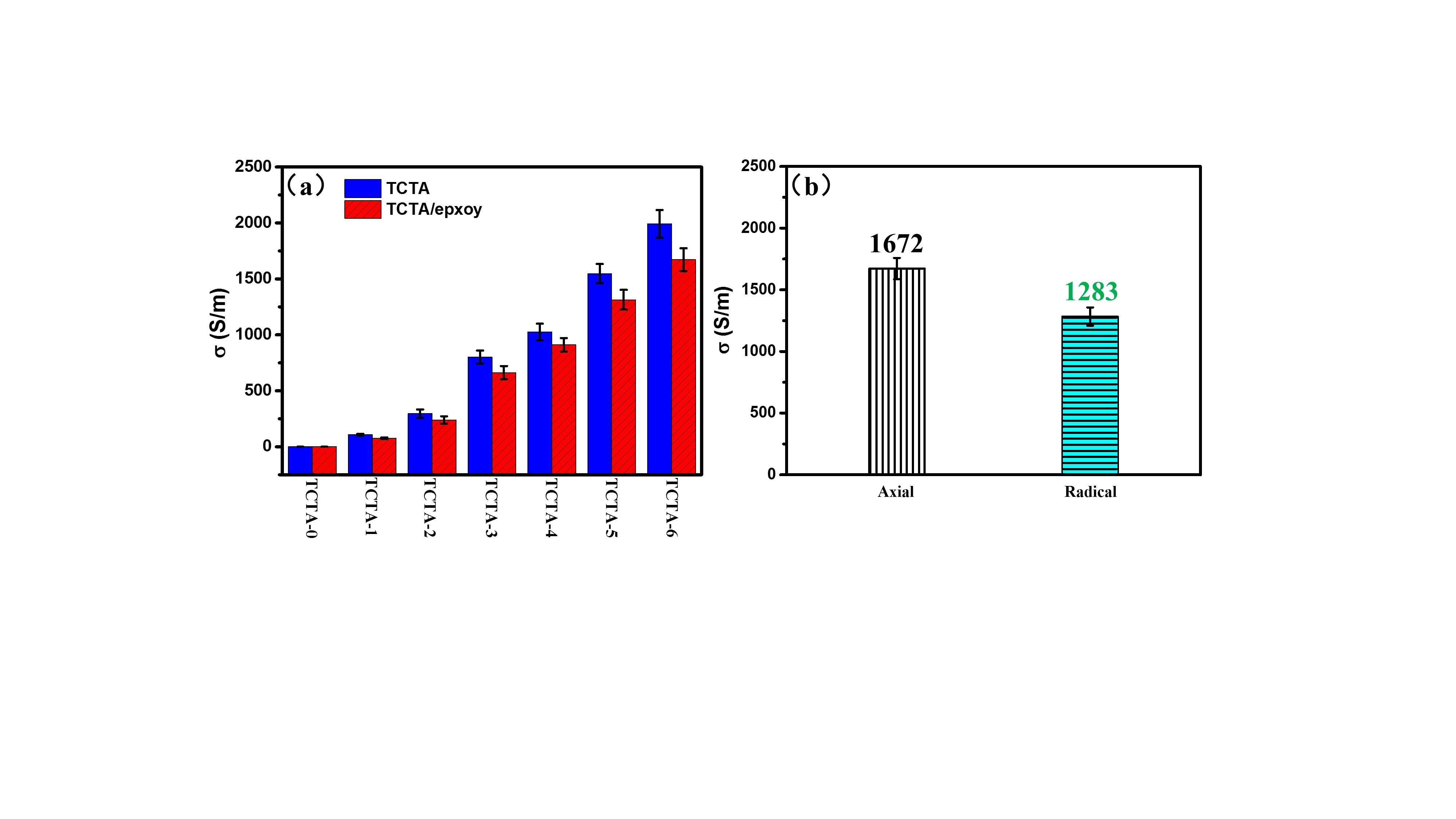


**Figure S6.** (a) Contrast of σ values for TCTA and TCTA/epoxy nanocomposites; (b) σ values of the TCTA-6/epoxy nanocomposites in axial and radical direction.


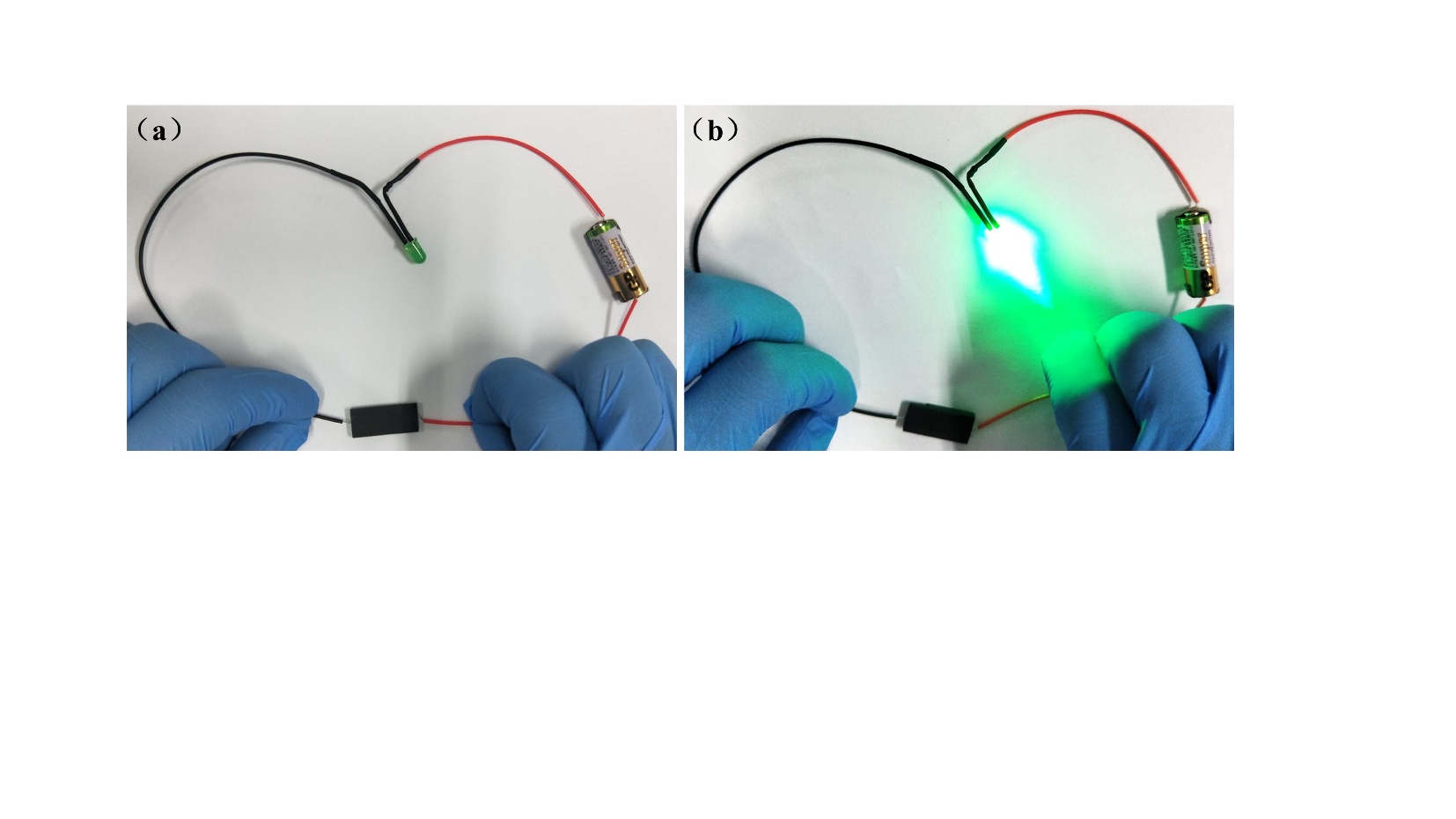


**Figure S7.** Digital photographs showing LED lamp at 3 V with (a) TCTA-0/epoxy and (b) TCTA-6/epoxy nanocomposites used as electrically conductive elements.

**
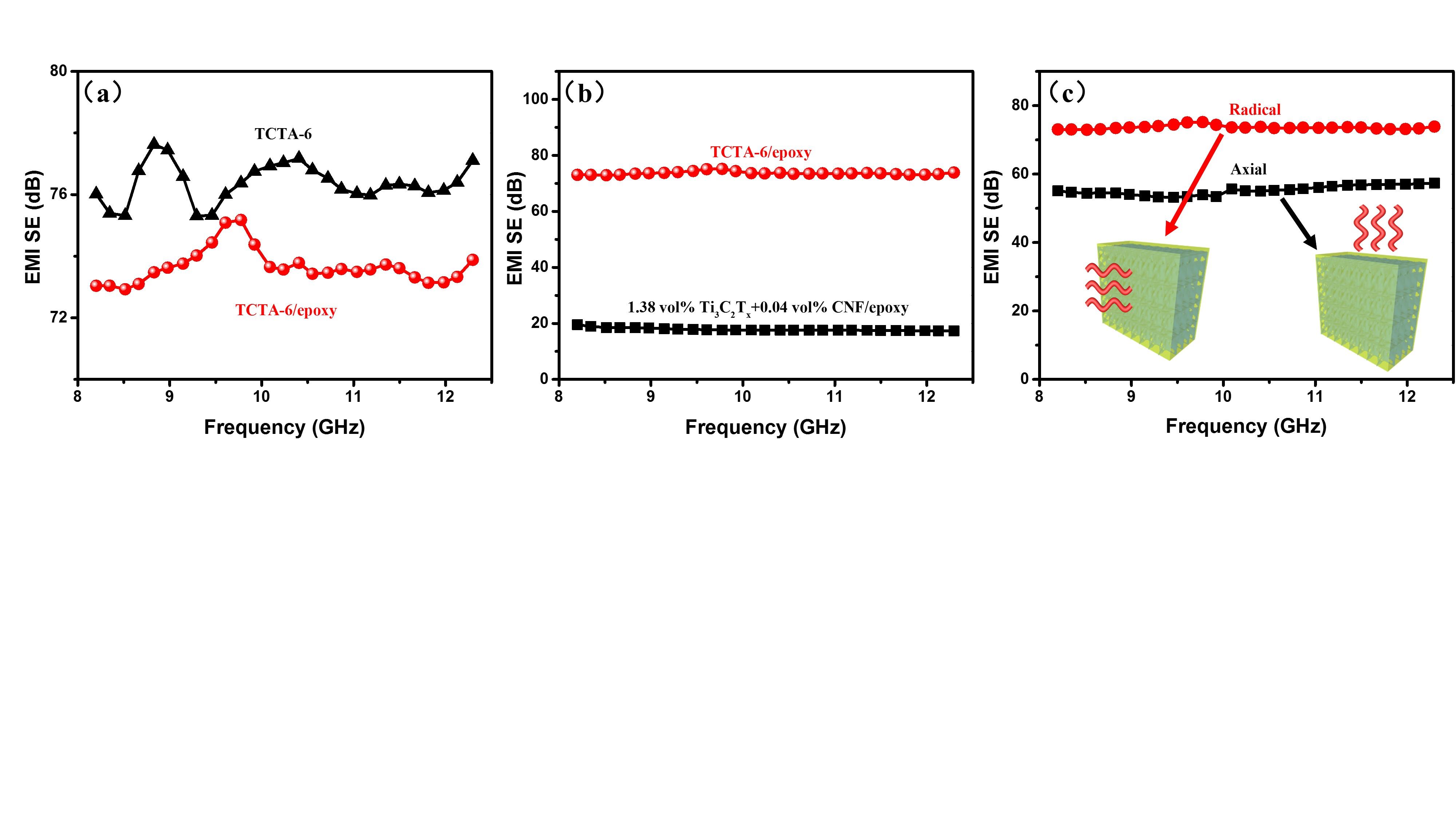
**

**Figure S8.** (a) Comparison of the EMI SE values between TCTA-6 and TCTA-6/epoxy nanocomposites; (b) Comparison of EMI SE values for TCTA-6/epoxy nanocomposites *vs* epoxy nanocomposites fabricated by blend-casting method; (c) EMI SE values of the TCTA-6/epoxy nanocomposites in axial and radical direction.

**
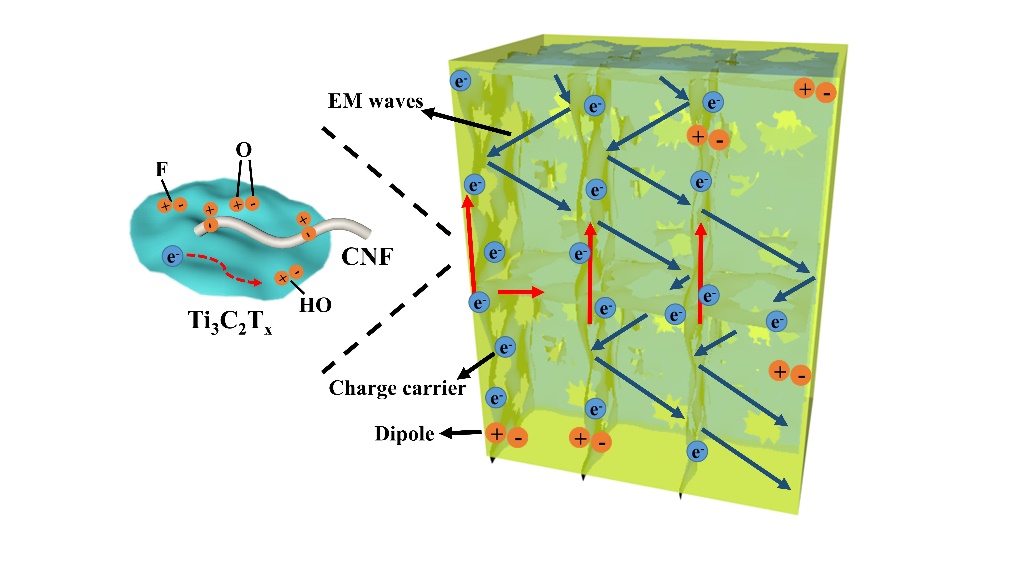
**

**Figure S9.** Interaction between EM waves and microstructures of the TCTA/epoxy nanocomposites.


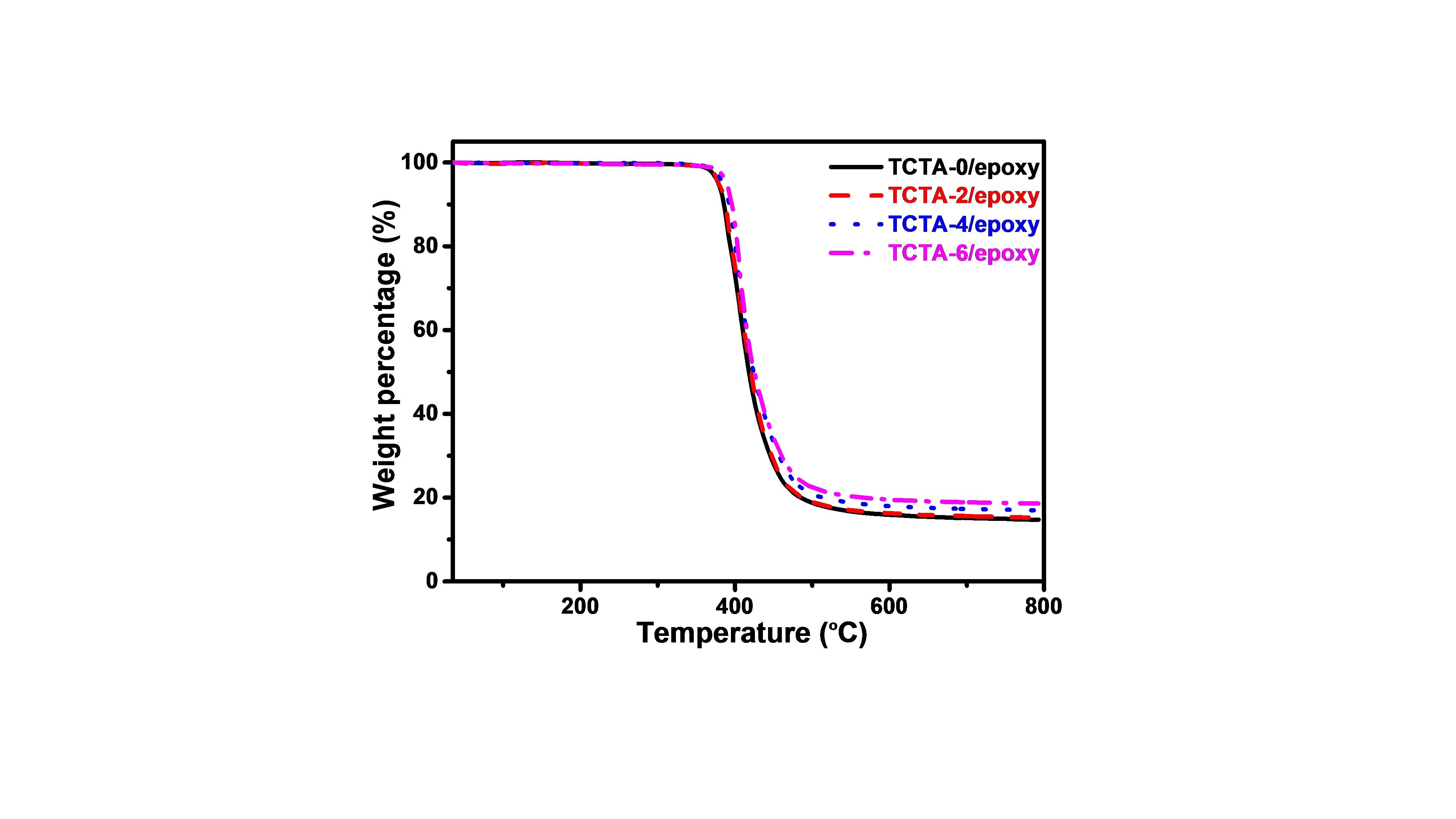


**Figure S10.** TGA curves of the TCTA/epoxy nanocomposites.

**Table S1.** Comparison of electrical conductivities for TCTA with different densities.

| **Samples** | **Density (mg/cm^3^)** | **Conductivity (S/m)** |
| --- | --- | --- |
| **TCTA-0** | 2.4 | 0.0186 |
| **TCTA-1** | 5.0 | 108 |
| **TCTA-2** | 9.6 | 296 |
| **TCTA-3** | 18.1 | 802 |
| **TCTA-4** | 26.8 | 1026 |
| **TCTA-5** | 36.1 | 1548 |
| **TCTA-6** | 45.0 | 1992 |

**Table S2.** Comparison of EMI SE values for TCTA/epoxy nanocomposites.

| **Samples** | **Ti_3_C_2_T_x_ content (vol %)** | **Total filler content (vol %)** | **Conductivity (S/m)** | **EMI SE (dB)** |
| --- | --- | --- | --- | --- |
| **TCTA-0/epoxy** | 0 | 0.04 | 8.6×10^-3^ | 7 |
| **TCTA-1/epoxy** | 0.13 | 0.17 | 76 | 22 |
| **TCTA-2/epoxy** | 0.28 | 0.32 | 240 | 34 |
| **TCTA-3/epoxy** | 0.54 | 0.58 | 663 | 46 |
| **TCTA-4/epoxy** | 0.82 | 0.86 | 911 | 56 |
| **TCTA-5/epoxy** | 1.11 | 1.15 | 1314 | 64 |
| **TCTA-6/epoxy** | 1.38 | 1.42 | 1672 | 74 |

**Table S3.** Comparison of EMI SE values of the TCTA/epoxy nanocomposites with other works.

| **Nanocomposites** | **Fillers Content** | **Conductivity** | **EMI SE** | **Thickness** | **SE/d** | **Frequency** | **Refs** |
| --- | --- | --- | --- | --- | --- | --- | --- |
|  | **vol%** | **S/m** | **dB** | **mm** | **dB/ mm** | **GHz** |  |
| **Ag nanowires/PS** | 2.50 | 1900 | 31.9 | 0.8 | 39.8 | 8.2-12.4 | ^[3]^ |
| **Ni Fiber/PES** | 7.00 | -- | 58 | 2.85 | 20.4 | 1-2 | ^[4]^ |
| **Al Flakes/PES** | 20 | -- | 39 | 2.9 | 13.5 | 1-2 | ^[5]^ |
| **rGO/PS** | 3.47 | 43.5 | 45.1 | 2.5 | 18 | 8.2-12.4 | ^[6]^ |
| **rGO/wax** | 20 | < 0.1 | 29 | 2 | 14.5 | 8.2-12.4 | ^[7]^ |
| **rGO/WPU** | 5.00 | 16.8 | 34 | 1 | 34 | 8.2-12.4 | ^[8]^ |
| **rGO/PEI** | 1.38 | 2.2×10^-3^ | 13 | 2.3 | 5.7 | 8-12 | ^[9]^ |
| **S-doped RGO/epoxy** | 7.5 | 33 | 24.5 | 2 | 12.3 | 12.4-18 | ^[10]^ |
| **Graphene foam/PDMS** | 0.36 | 200 | 20 | 1 | 20 | 8-12 | ^[11]^ |
| **TGO/PMMA** | 4.2 | 20 | 3.4 | 30 | 8.8 | 8-12 | ^[12]^ |
| **Aligned TGO foam/epoxy** | 0.36 | 980 | 32 | 4 | 8 | 8-12 | ^[13]^ |
| **MWCNT/PLLA** | 1.47 | 3.4 | 23 | 2.5 | 9.2 | 8.0-12.4 | ^[14]^ |
| **MWCNT/WPU** | 7.20 | 44.6 | 50 | 4.5 | 11.1 | 8.2-12.4 | ^[15]^ |
| **MWCNT/PP** | 7.50 | ≈10^-4^ | 35 | 1 | 35 | 8-12 | ^[16]^ |
| **SWCNT/PU** | 14.28 | 2.20×10^-2^ | 17 | 2 | 8.5 | 8.2-12.4 | ^[17]^ |
| **SWCNT/epoxy** | 10.53 | 0.2 | 28 | 2 | 14 | 8.2-12.4 | ^[18]^ |
|  | 10.53 | 20 | 20 | 1.5 | 13.3 | 0.5-1.5 | ^[19]^ |
| **SWCNT/PMMA** | 14.28 | 2 | 40 | 4.5 | 8.9 | 8-12 | ^[20]^ |
| **CNT sponge/epoxy** | 1.34 | 516 | 40 | 2 | 20 | 8-12 | ^[21]^ |
| **Carbon black/ABS** | 8.93 | 30 | 22 | 1.1 | 20 | 8.2-12.4 | ^[22]^ |
| **Expanded graphite/ SEBS** | 9.96 | 24 | 12 | 5 | 2.4 | 8.2-12.4 | ^[23]^ |
| **Carbon nanowires/ graphene/PDMS** | 13.03 | 340 | 36 | 1.6 | 22.5 | 8.2-12.4 | ^[24]^ |
| **TGO/Fe_3_O_4_/PS** | 4.13 | 21 | 30 | 4 | 7.5 | 8-12 | ^[25]^ |
| **RGO/Fe_3_O_4_/PVC** | 3.4 | 7.7×10^-4^ | 13 | 1.8 | 7.2 | 8.2-12 | ^[26]^ |
| **RGO/Fe_2_O_3_/PVA** | 10.76 | 3 | 20.3 | 0.36 | 56.3 | 8.2-12.4 | ^[27]^ |
| **Ti_3_C_2_T_x_/epoxy** | 5.6 | 105 | 41 | 2 | 20.5 | 8.2-12.4 | ^[28]^ |
| **Ti_3_C_2_T_x_/wax** | 16.88 | -- | 39 | 2 | 19.5 | 2-18 | ^[29]^ |
| **Ti_3_C_2_T_x_/C foam/epoxy** | 1.96 | 184 | 46 | 2 | 23 | 8.2-12.4 | ^[30]^ |
| **PS@Ti_3_C_2_T_x_** | 1.9 | 1081 | 62 | 2 | 31 | 8.2-12.4 | ^[31]^ |
| **Ti_3_C_2_T_x_/rGO/**  **epoxy** | 0.99 | 695.9 | 56.4 | 2 | 28.2 | 8.2-12.4 | ^[32]^ |
| ***TCTA/epoxy*** | ***0.85*** | ***911*** | ***56*** | ***2*** | ***28*** | ***8.2-12.4*** | ***This work*** |
|  | ***1.15*** | ***1314*** | ***64*** | ***2*** | ***32*** | ***8.2-12.4*** |  |
|  | ***1.42*** | ***1672*** | ***74*** | ***2*** | ***37*** | ***8.2-12.4*** |  |

**Table S4.** Thermal parameters of the TCTA/epoxy nanocomposites.

| **Samples** | **Weight loss temperature/^o^C** | | | ***T*_Heat-resistance index_*/^o^C** | **Residues (%)** |
| --- | --- | --- | --- | --- | --- |
|  | ***T*_5_** | ***T*_30_** | ***T*_50_** |  |  |
| **TCTA-0/epoxy** | 378.5 | 402.5 | 418.2 | 303.8 | 14.7 |
| **TCTA-2/epoxy** | 379.9 | 404.1 | 420.8 | 305.0 | 15.2 |
| **TCTA-4/epoxy** | 384.1 | 406.8 | 424.2 | 307.8 | 17.0 |
| **TCTA-6/epoxy** | 389.1 | 408.32 | 425.2 | 310.7 | 18.6 |

*Sample’s heat-resistance index is calculated by Equation S1.

*T_Heat-resistance index_*=0.49×[*T_5_*+0.6×(*T_30_*-*T_5_*)] (Equation S1)

*T_5_* and *T_30_* are corresponding decomposition temperature of 5% and 30% weight loss, respectively.

**References**

[1] M. Alhabeb, K. Maleski, B. Anasori et al., "Guidelines for synthesis and processing of two-dimensional titanium carbide (Ti_3_C_2_T_x_ MXene)," Chemistry of Materials, vol. 29, no. 18, pp. 7633-7644, 2017.

[2] X. Xie, M. Q. Zhao, B. Anasori et al., "Porous heterostructured MXene/carbon nanotube composite paper with high volumetric capacity for sodium-based energy storage devices," Nano Energy, vol. 26, pp. 513-523, 2016.

[3] M. Arjmand, A. A. Moud, Y. Li, and U. Sundararaj, "Outstanding electromagnetic interference shielding of silver nanowires: Comparison with carbon nanotubes," RSC Advances, vol. 5, no. 70, pp. 56590-56598, 2015.

[4] X. Shui, and D. Chung, "Nickel filament polymer-matrix composites with low surface impedance and high electromagnetic interference shielding effectiveness," Journal of Electronic Materials, vol. 26, no. 8, pp. 928-934, 1997.

[5] L. I. Lin, Chuang, and D. L. D., "Electrical and mechanical properties of electrically conductive polyethersulfone composites," Composites, vol. 25, no. 3, pp. 215-224, 1994.

[6] D. X. Yan, H. Pang, B. Li et al., "Structured reduced graphene oxide/polymer composites for ultra-efficient electromagnetic interference shielding," Advanced Functional Materials, vol. 25, no. 4, pp. 559-566, 2015.

[7] B. Wen, X. Wang, W. Cao et al., "Reduced graphene oxides: The thinnest and most lightweight materials with highly efficient microwave attenuation performances of the carbon world," Nanoscale, vol. 6, no. 11, pp. 5754-5761, 2014.

[8] S. T. Hsiao, C. C. M. Ma, W. H. Liao et al., "Lightweight and flexible reduced graphene oxide/water-borne polyurethane composites with high electrical conductivity and excellent electromagnetic interference shielding performance," ACS Applied Materials & Interfaces, vol. 6, no. 13, pp. 10667-10678, 2014.

[9] J. Ling, W. Zhai, W. Feng, B. Shen, and G. Z. Wen, "A facile preparation of lightweight microcellular polyetherimide/graphene composites foams for electromagnetic interference (EMI) shielding," ACS Applied Materials & Interfaces, vol. 5, no. 7, pp. 2677-2684, 2013.

[10] F. Shahzad, S. Yu, P. Kumar et al., "Sulfur doped graphene/polystyrene nanocomposites for electromagnetic interference shielding," Composite Structures, vol. 133, pp. 1267-1275, 2015.

[11] Z. Chen, C. Xu, C. Ma, W. Ren, and H. M. Cheng, "Lightweight and flexible graphene foam composites for high-performance electromagnetic interference shielding," Advanced Materials, vol. 25, no. 9, pp. 1296-1300, 2013.

[12] S. Barwich, and J. N. Coleman, "Yielding and flow of highly concentrated, few-layer graphene suspensions," Soft Matter, vol. 11, no. 16, pp. 3159-3164, 2015.

[13] X. H. Li, X. Li, K. N. Liao et al., "Thermally annealed anisotropic graphene aerogels and their electrically conductive epoxy composites with excellent electromagnetic interference shielding efficiencies," ACS Applied Materials & Interfaces, vol. 8, no. 48, pp. 33230-33239, 2016.

[14] T. Kuang, L. Chang, C. Feng et al., "Facile preparation of lightweight high-strength biodegradable polymer/multi-walled carbon nanotubes nanocomposite foams for electromagnetic interference shielding," Carbon, vol. 105, pp. 305-313, 2016.

[15] Z. Zeng, H. Jin, M. Chen et al., "Lightweight and anisotropic porous MWCNT/WPU composites for ultrahigh performance electromagnetic interference shielding," Advanced Functional Materials, vol. 26, no. 2, pp. 303-310, 2016.

[16] M. H. Al-Saleh, and U. Sundararaj, "Electromagnetic interference shielding mechanisms of CNT/polymer composites," Carbon, vol. 47, no. 7, pp. 1738-1746, 2009.

[17] Z. Liu, B. Gang, H. Yi et al., "Reflection and absorption contributions to the electromagnetic interference shielding of single-walled carbon nanotube/polyurethane composites," Carbon, vol. 45, no. 4, pp. 821-827, 2007.

[18] H. Yi, L. Ning, Y. Ma et al., "The influence of single-walled carbon nanotube structure on the electromagnetic interference shielding efficiency of its epoxy composites," Carbon, vol. 45, no. 8, pp. 1614-1621, 2007.

[19] N. Li, Y. Huang, F. Du et al., "Electromagnetic interference (EMI) shielding of single-walled carbon nanotube epoxy composites," Nano Letters, vol. 6, no. 6, pp. 1141-1145, 2006.

[20] N. C. Das, Y. Liu, K. Yang et al., "Single-walled carbon nanotube/poly (methyl methacrylate) composites for electromagnetic interference shielding," Polymer Engineering & Science, vol. 49, no. 8, pp. 1627-1634, 2009.

[21] Y. Chen, H. B. Zhang, Y. Yang et al., "High-performance epoxy nanocomposites reinforced with three-dimensional carbon nanotube sponge for electromagnetic interference shielding," Advanced Functional Materials, vol. 26, no. 3, pp. 447-455, 2016.

[22] M. H. Al-Saleh, W. H. Saadeh, and U. Sundararaj, "EMI shielding effectiveness of carbon based nanostructured polymeric materials: A comparative study," Carbon, vol. 60, no. 12, pp. 146-156, 2013.

[23] S. Kuester, C. Merlini, G. M. O. Barra et al., "Processing and characterization of conductive composites based on poly(styrene-b-ethylene-ran-butylene-b-styrene) (SEBS) and carbon additives: A comparative study of expanded graphite and carbon black," Composites Part B Engineering, vol. 84, pp. 236-247, 2016.

[24] K. Luo, X. Yin, M. Han et al., "Macroscopic bioinspired graphene sponge modified with in-situ grown carbon nanowires and its electromagnetic properties," Carbon, vol. 111, pp. 94-102, 2017.

[25] Y. Chen, Y. Wang, H. B. Zhang et al., "Enhanced electromagnetic interference shielding efficiency of polystyrene/graphene composites with magnetic Fe_3_O_4_ nanoparticles," Carbon, vol. 82, pp. 67-76, 2015.

[26] K. Yao, G. Jiang, N. Tian et al., "Flammability properties and electromagnetic interference shielding of PVC/graphene composites containing Fe_3_O_4_ nanoparticles," RSC Advances, vol. 5, no. 40, pp. 31910-31919, 2015.

[27] B. Yuan, C. Bao, X. Qian et al., "Design of artificial nacre-like hybrid films as shielding to mitigate electromagnetic pollution," Carbon, vol. 75, no. 2, pp. 178-189, 2014.

[28] L. Wang, L. Chen, P. Song et al., "Fabrication on the annealed Ti_3_C_2_T_x_ MXene/epoxy nanocomposites for electromagnetic interference shielding application," Composites Part B: Engineering, vol. 171, pp. 111-118, 2019.

[29] X. Liu, J. Wu, J. He, and L. Zhang, "Electromagnetic interference shielding effectiveness of titanium carbide sheets," Materials Letters, vol. 205, pp. 261-263, 2017.

[30] L. Wang, H. Qiu, P. Song et al., "3D Ti_3_C_2_T_x_ MXene/C hybrid foam/epoxy nanocomposites with superior electromagnetic interference shielding performances and robust mechanical properties," Composites Part A: Applied Science and Manufacturing, vol. 123, pp. 293-300, 2019.

[31] R. Sun, H. B. Zhang, J. Liu et al., "Highly conductive transition metal carbide/carbonitride(MXene)@polystyrene nanocomposites fabricated by electrostatic assembly for highly efficient electromagnetic interference shielding," Advanced Functional Materials, vol. 27, no. 45, article 1702807, 2017.

[32] S. Zhao, H. B. Zhang, J. Q. Luo et al., "Highly electrically conductive three-dimensional Ti_3_C_2_T_x_ MXene/reduced graphene oxide hybrid aerogels with excellent electromagnetic interference shielding performances," ACS Nano, vol. 12, no. 11, pp. 11193-11202, 2018.
